# Perception of quality of care of patients with potentially severe diseases evaluated at a distinct quick diagnostic delivery model: a cross-sectional study

**DOI:** 10.1186/s12913-015-1070-2

**Published:** 2015-09-30

**Authors:** Carmen Sanclemente-Ansó, Albert Salazar, Xavier Bosch, Cristina Capdevila, Amparo Giménez-Requena, Beatriz Rosón-Hernández, Xavier Corbella

**Affiliations:** Department of Internal Medicine, Bellvitge University Hospital, University of Barcelona, Biomedical Research Institute (IDIBELL), Consultas Externas, Area de Gestión Administrativa, c/Feixa Llarga s/n, 08907-L’Hospitalet de Llobregat, Barcelona, Spain; Emergency Department, Bellvitge University Hospital, Department of Medicine, University of Barcelona, Biomedical Research Institute (IDIBELL), L’Hospitalet de Llobregat, Barcelona, Spain; Department of Internal Medicine, Hospital Clinic, University of Barcelona, Institut d’Investigacions Biomèdiques August Pi i Sunyer (IDIBAPS), c/Villarroel 170, 08036 Barcelona, Spain; Department of Quality, Bellvitge University Hospital, University of Barcelona, Biomedical Research Institute (IDIBELL), L’Hospitalet de Llobregat, Barcelona, Spain; Global Institute of Public Health and Health Policy, School of Medicine, International University of Catalonia, Barcelona, Spain

**Keywords:** Quick diagnosis unit, Perception of quality, Recommendation, Satisfaction, Outpatients, Personal interaction, Clinical information, Waiting times, Physical environment, Regression coefficient

## Abstract

**Background:**

Although hospital-based outpatient quick diagnosis units (QDU) are an increasingly recognized cost-effective alternative to hospitalization for the diagnosis of potentially serious diseases, patient perception of their quality of care has not been evaluated well enough. This cross-sectional study analyzed the perceived quality of care of a QDU of a public third-level university hospital in Barcelona.

**Methods:**

One hundred sixty-two consecutive patients aged ≥ 18 years attending the QDU over a 9-month period were invited to participate. A validated questionnaire distributed by the QDU attending physician and completed at the end of the first and last QDU visit evaluated perceived quality of care using six subscales.

**Results:**

Response rate was 98 %. Perceived care in all subscales was high. Waiting times were rated as ‘short’/’very short’ or ‘better’/’much better’ than expected by 69–89 % of respondents and physical environment as ‘better’/’much better’ than expected by 94–96 %. As to accessibility, only 3 % reported not finding the Unit easily and 7 % said that frequent travels to hospital for visits and investigations were uncomfortable. Perception of patient–physician encounter was high, with 90–94 % choosing the positive extreme ends of the clinical information and personal interaction subscales items. Mean score of willingness to recommend the Unit using an analogue scale where 0 was ‘never’ and 10 ‘without a doubt’ was 9.5 (0.70). On multivariate linear regression, age >65 years was an independent predictor of clinical information, personal interaction, and recommendation, while age 18–44 years was associated with lower scores in these subscales. No schooling predicted higher clinical information and recommendation scores, while university education had remarkable negative influence on them. Having ≥4 QDU visits was associated with lower time to diagnosis and recommendation scores and malignancy was a negative predictor of time to diagnosis, clinical information, and recommendation.

**Discussion:**

It is worthy of note that the questionnaire evaluated patient perception and opinions of healthcare quality including recommendation rather than simply satisfaction. It has been argued that perception of quality of care is a more valuable approach than  satisfaction. In addition to embracing an affective dimension, satisfaction appears more dependent on patient expectations than is perception of quality.

**Conclusions:**

While appreciating that completing the questionnaire immediately after the visit and its distribution by the QDU physician may have affected the results, scores of perceived quality of care including recommendation were high. There were, however, significant differences in several subscales associated with age, education, number of QDU visits, and diagnosis of malignant vs. benign condition.

**Electronic supplementary material:**

The online version of this article (doi:10.1186/s12913-015-1070-2) contains supplementary material, which is available to authorized users.

## Background

In Spain and other countries, patients needing diagnostic examinations for potentially serious diseases have traditionally occupied acute beds, without requiring actual therapy [1–4]. While it has been reported that up to 20 % of Spanish patients hospitalized in general internal medicine wards could have be studied as outpatients [2, 5–9], European studies have shown that inappropriate use of hospital beds surpasses 20 % through diverse specialties [3, 10–13]. A study by Campbell *et al.* revealed that, if given the choice, 60 % of physicians would contemplate an alternative to hospitalization for these patients and that 70 % of patients would choose not to be admitted for undergoing diagnostic examinations [11].

Lengthy waits in diagnostic workups owe to insufficiencies in outpatient services, with diagnosis in such settings being unfeasible, even when quick investigations for suspected cancer are necessary [14–16]. Because current referral procedures for diagnosis and specialized care in primary healthcare centers (PCCs) are prolonged, especially in public health systems such as the Spanish one, PCC physicians commonly refer patients with suspected serious diseases to the emergency department (ED) with the hope to gain quick access to examinations via hospitalization [12, 13, 17, 18]. Alternatively, patients are free to pay for diagnostic tests such as a CT scan or MRI using a private provider [19].

These shortcomings have led to the design of alternatives to conventional hospitalization for medical disorders, more recently hospital-based outpatients quick diagnosis units (QDUs). Although still little known today, this type of units, run by internists, nurses, and secretarial staff, have been established in Europe, and they have been principally studied in Spain [1, 2, 4, 12–15, 17, 18, 20–24]. The advantages over conventional hospitalization are numerous: in addition to ensuring an early diagnosis, QDUs avoid hospital-related morbidity, decrease ED referrals from PCCs and decongestion overcrowded ED, and help decrease unnecessary health costs of traditional hospitalization without lowering the quality of diagnostic practice and patient care [2, 12, 14, 17, 18, 21–24]. Yet for QDUs to succeed, the following requirement are expected to be met: 1) clear pre-established referral criteria; 2) patients should be well enough to attend outpatient appointments for visits and diagnostic tests; 3) their first visit has to occur as soon as possible after referral; and 4) they should have preferential access to diagnostic tests [12, 13].

Because a critical element of this innovative approach is to cause a minimal disruption of the patient’s daily life, patient opinion on the QDU model would be expected to be high. However, no study has analyzed in detail patient perception of or satisfaction with the quality of care delivered by these units. Indeed, increasing interest in taking into account patient opinions of the quality of healthcare services has led to the elaboration of many measurement tools (i.e. questionnaires) meant to measure patient satisfaction, perception, or experience [25, 26]. Such surveys are important not only to guide subsequent service delivery but also because they may impact clinical outcomes [27, 28]. While surveys have been conducted across a broad range of clinical settings (e.g. hospital outpatients clinics, inpatients, and primary care) [29], only two studies, one published in a Spanish-language [2] and other in an English-language journal [21] have reported some general information about satisfaction with QDU, as a part of a comprehensive descriptive analysis of the functioning and usefulness of these units.

The purpose of this study was to evaluate, using a validated questionnaire, patient perception of quality of care of a QDU of a public third-level university hospital in Barcelona (Spain) and to analyze the relationship of perceived quality to background patient factors.

## Methods

### Design, setting and participants

One hundred sixty-two consecutive patients aged ≥ 18 years attending the QDU over a 9-month period (from 6 March 2012 to 7 December 2012, with about 18 patients on average per month) were invited to participate in a cross-sectional study.

The QDU is part of the internal medicine department of the Bellvitge University Hospital, Barcelona, Spain, a third-level public hospital with 906 acute beds serving a reference population of 343,200. The hospital is a reference center for more than 2 million people for high technology processes, and is equipped with all medical and surgical specialties except obstetrics and pediatrics. The QDU comprises an internal medicine specialist and a nurse, who work for 7 hours a day, 2 days a week in the Unit, with coordinated support from other specialists. The QDU has a consulting room and a waiting room for patients and families. Patients are referred from the ED, PCCs, and specialized outpatient clinics. Predefined referral criteria include peripheral lymphadenopathies, anemia (with or without symptoms) with hemoglobin level <9 g/l, unintentional weight loss (loss of > 10 % of body weight during > 6 weeks), unexplained febrile syndrome (temperature > 38 °C during > 2 weeks), lung and/or pleural radiologic abnormalities, suspected tumor, abdominal mass, unexplained dysphagia, unexplained persistent severe abdominal pain, persistent change in bowel rhythm (>1 month), ascites in non-cirrhotic patients, hepatosplenomegaly, abnormalities in liver function tests, and non-obstructive jaundice [20]. Referrals to QDU are made by the hospital computer system, phone calls or e-mail. The QDU attending physician determines the appropriateness of referrals. The care protocol consists of an urgent first visit followed by preferential programming of diagnostic tests and subsequent visits until a diagnosis is made. In addition to the diagnostic tests typical of a third-level hospital in Spain, there is a dedicated circuit for the evaluation of lymphadenopathy. In particular, in cases of suspected malignant lymphadenopathy, fine-needle aspiration cytology is performed immediately at the time of first patient encounter, with cytological results available in 30 minutes; in addition, since November 2011, immunocytochemical studies, especially flow cytometry, are available for the diagnosis of lymphomas.

### Questionnaire

The questionnaire used (see Additional file 1) was an adaptation of the Patient Satisfaction Survey, originally developed by the Association of Urologists of Columbia (USA) for evaluating the perception of quality of care by patients attended at outpatient urologic clinics [30]. Previous Spanish studies using the original version, translated into Spanish, have been validated for hospital medical outpatients in Spain [31, 32]. These studies appeared to indicate that the questionnaire could be highly suitable for QDU patients. While the questionnaire used in the current study used the same subscales and items as the original version [30], it was adapted to QDU patients by incorporating a specific question about time to diagnosis. The Department of Quality of Bellvitge University Hospital internally validated the questionnaire through a pilot study. The analysis of the psychometric properties of the different subscales showed favorable evidence concerning their reliability and validity. For instance, test reliability revealed a high level of internal consistency for the six subscales with a Cronbach's alpha ranging from 0.77 to 0.89.

The QDU attending physician personally invited all patients to complete the questionnaire in two stages: at the end of the first QDU visit and at the end of the last visit. In the first stage (questions No. 1–8 of the questionnaire: see Additional file 1), patients were asked questions about demographic and education characteristics, previous use of outpatient clinics, previous hospitalizations at Bellvitge University Hospital, ease to find the Unit, name of the QDU physician and nurse, and perceived waiting time on the waiting room. In the second stage (questions No. 9–20 of the questionnaire: see Additional file 1), patients were asked, among others, questions about personal interaction, visit duration, perception of physical environment and clinical information, time to diagnosis, degree of recommendation, and awareness of final diagnosis. Patients having only 1 visit were asked to complete the full questionnaire at the end of this visit.

The own QDU physician distributed the questionnaire with an envelope at the end of first visit to every consecutive patient attending the QDU during the survey period. After completing the first part of the questionnaire, patients returned it to the administrative staff before leaving the facility. At time of last visit, the administrative staff distributed the partly filled forms within their envelopes to patients on arrival and the QDU physician reminded them, at the end of this visit, to complete and return them to the administrative staff before leaving the facility.

There was sufficient time and a private space to answer anonymously at the two stages. Each questionnaire form and its corresponding envelope were marked with a unique identifying number but we assured patients that anonymity would be maintained.

### Measurements

Perceived quality of care was assessed with six subscales containing a total of 15 items. These subscales (and number of items used) were: 1) waiting times (three items); 2) physical environment (three items); 3) accessibility (two items); 4) provider clinical information (four items); 5) personal interaction (two items); and 6) recommendation (one item). Five-point scales were used to score the items composing the waiting times and physical environment subscales. In the ‘Duration of visit’ item of the waiting times subscale of the original administered questionnaire, a higher score meant ‘better’, while in the other two items (i.e. ‘time on the waiting room’ and ‘time to diagnosis’) a higher score meant ‘worse’. Accordingly, to aid comparability, these 2 items were reverse-coded. With regard to the clinical information and personal interaction subscales, although items contained 5 answering options, the last option (code 5) was excluded from the calculation of means as it stands for ‘not sure/don’t remember’. Therefore, these two subscales were indeed 4-point scales. The average score for the physical environment, clinical information, and personal interaction subscales was equal to the average of responses to each specific item, then dividing the resulting total score by the number of items of each subscale. Because of the distinctive connotation of the times intended to be perceived in the waiting times subscale, the mean scores were calculated separately for each of the items of this subscale. While the accessibility subscale was assessed with a single dichotomous (‘yes/no’) variable, the recommendation subscale was measured with the question ‘Would you recommend this QDU to a relative with the same disease, in case of need?’ A visual analogue scale ranging from 0 (‘never’) to 10 (‘without a doubt’) was used. The Unit was considered recommendable when it was rated ≥7 and not recommendable or doubtful when it was rated <7. The questionnaire also included three questions that might implicitly reflect perception of, or overall satisfaction with, quality of care: 1) the question ‘Would you have preferred to have been admitted to hospital to study your disease?’ was assessed with a single dichotomous (‘yes/no’) variable; 2) the open question ‘What can we do better?’ offered the option of writing a free commentary; and 3) an open question with a dichotomous (‘yes/no’) answer intended to determine whether the respondents had experienced any ‘problem’ with different aspects of the healthcare process.

Response rates and missing values were checked including no responses and the answering option ‘not sure/don't’ remember’ (code 5 in the clinical information and personal interaction subscales). We intended to exclude from the analyses any item found to have missing value rates ≥10 % [33].

In addition to the information contained in the questionnaires, data were gathered on monthly income, referral sources, mean wait times from referral to first visit, clinical reasons for consultation, mean number of visits, mean time to diagnosis, final diagnoses, and onwards referrals.

### Ethical considerations

The Research Ethics Committee of Bellvitge University Hospital approved this study. At the time of first visit, participants were given detailed information about the study and written informed consent was obtained from all them.

### Statistical analyses

Descriptive statistics were calculated to determine patient characteristics. Categorical variables are expressed as absolute frequencies (%). Continuous variables are expressed as mean (standard deviation). Individual questionnaire items were analyzed using the chi-square test and overall subscale scores were analyzed using the *t*-test or Mann–Whitney test, as appropriate. An analysis of variance (ANOVA) was firstly conducted to determine how the mean scores of the different subscales varied by each patient characteristic that was used as a covariate. In order to adjust for confounders and to identify the independent predictors associated with the mean scores of subscales, independent variables that were marginally significant (*P* < 0.20) in bivariate analysis were included in a multivariate linear regression analysis. Predictive variables are expressed in terms of regression coefficients beta (*B*) with 95 % confidence intervals (CI). The level of statistical significance was set to *P* < 0.05. The statistical analyses were performed using SPSS software, version 21 (SPSS Inc., Chicago, Ill, USA).

## Results

A total of 159 outpatients participated in the study, representing a response rate of 98 %. Three (2 %) patients who did not participate stated that they did not have time to take the survey. Table 1 shows the characteristics of the respondents. Main referral sources were the ED (53 %) and PCCs (35 %). The mean (SD) age was 60.5 (17.42) years and 53 % were males. Forty percent of patients had primary education and 30 % had no schooling. The monthly household income was Euros 1201–1800 in 33 % of patients and Euros 901–1200 in 28 %. Thirty-five percent of patients had previously been admitted to our hospital. Mean number of QDU visits (including the first visit) was 2.2 (0.73). Eleven (7 %) patients required 1 visit, 84 (53 %) 2, 43 (27 %) 3, and 21 (13 %) 4 or more visits. The first 159 visits generated 187 successive visits (ratio successive/first =1.18). Clinical reasons for consultation in 65 % of patients were peripheral lymphadenopathies, unintentional weight loss, suspected masses, and anemia. Main diagnoses were malignancy (26 %), benign digestive disorders (11 %), reactive lymphadenopathy (7 %), and anemia (unrelated to malignancy) (5 %). The mean time to diagnosis was 12.2 (8.71) days.

**Table 1:** Characteristics of survey respondents at the quick diagnosis unit (*N* = 159)

Variable	N (%)	Mean (SD)
Age, years		60.5 (17.42)
18–44	24 (15)	
45–65	59 (37)	
> 65	76 (48)	
Sex		
Female	75 (47)	
Male	84 (53)	
Education		
University education	15 (9)	
Secondary or professional training	32 (20)	
Primary education	64 (40)	
No schooling	48 (30)	
Monthly household income, Euros^a^		1376 (185.11)
> 1800	29 (18)	
1201–1800	52 (33)	
901–1200	45 (28)	
≤ 900	33 (21)	
Previous visits at hospitals’ outpatients clinics	93 (59)	
Previous hospitalizations at this hospital	56 (35)	
Referral sources		
Emergency department	84 (53)	
Primary care centers	55 (35)	
Specialized outpatient clinics	6 (4)	
Other	14 (9)	
Wait time from referral to first visit, days^b^		3.6 (1.30)
Main reasons for consultation		
Peripheral lymphadenopathies	39 (25)	
Unintentional weight loss	24 (15)	
Suspicion/palpation of masses	22 (14)	
Anemia	19 (12)	
QDU visits, number		2.2 (0.73)
≤ 2	95 (60)	
3	43 (27)	
≥ 4	21 (13)	
Time to diagnosis, days^c^		12.2 (8.71)
Main diagnoses		
Malignancy	41 (26)	
Benign digestive disorders	18 (11)	
Reactive lymphadenopathy	11 (7)	
Anemia (unrelated to malignancy)	8 (5)	
Onwards referrals		
Primary care centers	83 (52)	
Specialized outpatient clinics	65 (41)	
Hospitalization	8 (5)	
Emergency department	2 (1)	
Other	1 (1)	

*SD* = standard deviation; *QDU* = quick diagnosis unit.

^a^Income of all household members, after tax deductions. Respondents had to choose one alternative from eight income ranges.

^b^From date of referral to date of first visit. Consecutive days, including weekends and holidays.

^c^From first QDU visit to definitive diagnosis (i.e. last visit)

Most patients knew the name of the QDU physician [*n* = 154 (97 %)] and the nurse [*n* = 129 (81 %)]. Table 2 shows the main results in terms of perceived quality of care in the full sample and displays both mean scores as well as relative frequencies. Rates of no responses, which are combined with ‘not sure/don’t remember’ responses in the clinical information and personal interaction subscales, are also shown. As to missing values, no item reached the 10 % cut-off level. Average missing values of all 15 items were 1.4 %, ranging from 0.6 to 1.9 %, suggesting no apparent difficulties in the comprehension of most questions. In addition, the specific rate of ‘not sure/don't’ remember’ responses of the clinical information and personal interaction subscales was 1.7 and 0.9 %, respectively.

**Table 2:** Patient scores and responses for individual items and subscales of perceived quality of care and recommendation

Subscales and items	Scores	N (%)
	Mean (SD)	
Waiting times		
Waiting time on the waiting room^a^	3.8 (0.78)	
(1–5) (1 = very excessive; 5 = very short)		
Scored 4–5		117 (74)
Scored 3		40 (25)
No response		2 (1)
Duration of visit	3.9 (0.73)	
(1–5) (1 = much worse than expected; 5 = much better than expected)		
Scored 4–5		142 (89)
Scored 2–3		14 (9)
No response		3 (2)
Time to diagnosis^a^	3.7 (0.82)	
(1–5) (1 = very excessive; 5 = very short)		
Scored 4–5		109 (69)
Scored 2–3		49 (31)
No response		1 (1)
Physical environment	4.5 (0.61)	
(1–5) (1 = much worse than expected; 5 = much better than expected)		
Consultation office temperature	4.6 (0.55)	
Scored 4–5		151 (95)
Scored 2–3		5 (3)
No response		3 (2)
Waiting room noise	4.5 (0.61)	
Scored 4–5		150 (94)
Scored 2–3		6 (4)
No response		3 (2)
Consultation office cleanliness	4.7 (0.52)	
Scored 4–5		153 (96)
Scored 2–3		4 (3)
No response		2 (1)
Clinical information	3.8 (0.66)	
(1–4) (1 = never; 4 = always)		
Meaning of disease	3.8 (0.72)	
Scored 4		145 (91)
Scored 2–3		10 (6)
Not sure/don't’ remember/No response		4 (3)
Diagnostic tests	3.9 (0.62)	
Scored 4		147 (92)
Scored 2–3		6 (4)
Not sure/don't’ remember/No response		6 (4)
Risks of diagnostic tests and treatment	3.8 (0.67)	
Scored 4		144 (91)
Scored 2–3		9 (6)
Not sure/don't’ remember/No response		6 (4)
Instructions after discharge	3.9 (0.63)	
Scored 4		149 (94)
Scored 2–3		7 (4)
Not sure/don't’ remember/No response		3 (2)
Personal interaction	3.7 (0.56)	
(1–4) (1 = never; 4 = always)		
Kindness of healthcare staff	3.8 (0.50)	
Scored 4		145 (91)
Scored 2–3		11 (7)
Not sure/don't’ remember/No response		3 (2)
Help of healthcare staff when needed	3.7 (0.55)	
Scored 4		143 (90)
Scored 2–3		11 (7)
Not sure/don't’ remember/No response		5 (3)
Accessibility		149 (94)
Found the unit easily		
Yes		151 (95)
No		5 (3)
No response		3 (2)
Found uncomfortable to travel frequently to hospital		
Yes		11 (7)
No		146 (92)
No response		2 (1)
Willingness to recommend the Unit	9.5 (0.70)	
(0–10) (0 = never; 10 = without a doubt)		
Rated < 7		6 (4)
Rated ≥ 7		151 (95)
No response		2 (1)

*SD* = standard deviation.

^a^Reverse-coded for the purpose of the analysis

Waiting time on the waiting room for the first visit was perceived as ‘short’ or ‘very short’ by 74 % of respondents and ’just right’ by 25 %, with none perceiving it as ’excessive’. While duration of visit was perceived as ’better’ or ’much better than expected’ by 89 % of respondents, ’as expected’ by 7 % and ’worse than expected’ by 2 %, time to diagnosis was considered ’short’ or ’very short’ by 69 %, ’just right’ by 25 % and ’excessive’ by 6 %. No patient chose the option ‘very excessive’ or ’much worse than expected’ for any item of this subscale. The mean score of the physical environment subscale was 4.5 (0.61), with 94–96 % of respondents rating the three constituting items as ’better’ or ’much better than expected’. Apart from 2 (1 %) patients who rated the waiting room noise as ’worse than expected’, ’as expected’ was the option selected by the remaining respondents for the 3 items of this subscale (3 % for each item) and none chose the response ‘much worse than expected’ for any item. As to perceived clinical information, its mean score was 3.8 (0.66), with answering option No. 3 being ‘usually’ and No. 4, ‘always’. Specifically, 91 % of respondents reported that they ‘always‘ received clear-cut information on what their disease involved, and 94 % said that they ‘always‘ received clear-cut information on instructions to follow after discharge. Similarly, ‘always’ was the response selected for information on forthcoming diagnostic tests and for information on the risks of diagnosis and treatment by 92 and 91 % of patients, respectively. While ‘usually’ was the most common answer after ‘always’ for all items of this subscale, 3 (2 %) respondents answered ‘rarely’ for information about meaning of disease, and 3 (2 %) also chose ‘rarely’ for information about risks of diagnosis and treatment. No individual responded ‘never’. The mean score of the personal interaction subscale was 3.7 (0.56), with answering choice No. 3 being ‘usually’ and No. 4, ‘always’. In particular, 91 % of respondents reported that healthcare staff ‘always‘ treated them with kindness, and 90 % said that healthcare staff ‘always‘ did their best to help when needed. While the ‘usually’ response was chosen by 9 (6 %) patients for the item ‘kindness’ of healthcare staff and by 8 (5 %) for the item ‘help’ of healthcare staff, ‘rarely’ was selected by 2 (1 %) and by 3 (2 %) respondents for the former and latter items, respectively. No individual answered ‘never’. With regard to accessibility, only 3 % of respondents reported that they did not find the Unit easily and 7 % said that frequent travels to hospital for QDU visits and diagnostic tests were uncomfortable (Table 2). The mean score of the recommendation subscale was 9.5 (0.70). The Unit was considered recommendable (score ≥7) by 95 % of respondents and not recommendable or doubtful (score <7) by 4 %. Of note, 129 (81 %) respondents scored 10/10 and none scored <5/10.

At the end of the QDU evaluation, only 6 (4 %) patients did not know their diagnosis, of whom 4 had no schooling and 2 had primary education. Furthermore, while 149 (94 %) patients said that they would not have preferred hospitalization to study their disease, the opposite was true for 6 (4 %), and 4 (3 %) did not respond. The most frequent answer to the question ‘What can we do better?’ was ‘nothing’ in 134 (84 %) respondents, and the remaining did not write any commentary. Finally, 6 (4 %) patients stated that they had experienced some ‘problem’ with the process. While 4 (3 %) reported that they were not given accurate information to locate the Unit easily, 2 (1 %) said that time to diagnosis was too long.

Tables 3 and 4 show the mean scores of the physical environment, waiting times, clinical information, personal interaction, and recommendation subscales according to patient characteristics on ANOVA. Scores did not differ by respondents’ sex, monthly income, and previous hospitalization. However, there were some significant differences for the rest of variables analyzed. Older respondents, and especially those aged > 65 years, had a better perception of visit duration, time to diagnosis, clinical information, and personal interaction, and they also reported higher recommendation scores. In addition, respondents with no schooling and primary education scored better than those with higher education levels the same items and subscales as older respondents except personal interaction. Patients having ≥4 QDU visits scored time to diagnosis and recommendation worse than those with 3 and especially ≤2 visits. When final diagnoses were dichotomized into malignant and non-malignant conditions, patients with malignancy scored time to diagnosis, clinical information, and recommendation worse than those with benign conditions (Tables 3 and 4). Finally, regarding accessibility, among the 11 (7 %) respondents who said that frequent travels to hospital were uncomfortable, 9 (12 %) were older than 65 years and 2 (3 %) were aged 37 and 52 years. The 5 (3 %) respondents who did not find the Unit easily were all older subjects: 3 were older than 65 years and 2 were aged 62 and 63 years. Reported answers in the accessibility subscale did not differ by other respondents’ variables.

**Table 3:** Mean scores of physical environment and waiting times subscales according to patient characteristics

Variable	Physical environment	*P* value	Waiting times					
			For visit^a^	*P* value	Visit duration	*P* value	Time to diagnosis^a^	*P* value
Age, years		0.15		0.36		**0.006**		**0.001**
18–44	4.4 (0.72)		3.7 (0.84)		3.5 (0.86)		3.2 (0.93)	
45–65	4.5 (0.65)		3.8 (0.80)		3.7 (0.78)		3.5 (0.82)	
> 65	4.6 (0.57)		3.8 (0.76)		4.1 (0.71)		3.9 (0.79)	
Sex		0.18		0.20		0.19		0.28
Female	4.4 (0.71)		3.7 (0.82)		3.8 (0.77)		3.8 (0.81)	
Male	4.6 (0.63)		3.9 (0.77)		4.0 (0.70)		3.7 (0.78)	
Education		0.10		0.09		**0.01**		**0.04**
University education	4.4 (0.81)		3.6 (0.88)		3.6 (0.91)		3.4 (0.94)	
Secondary/PT	4.4 (0.75)		3.7 (0.81)		3.7 (0.83)		3.5 (0.84)	
Primary education	4.5 (0.63)		3.8 (0.78)		4.0 (0.70)		3.8 (0.71)	
No schooling	4.7 (0.60)		3.9 (0.79)		4.1 (0.74)		3.8 (0.77)	
Monthly income, Euros		0.33		0.22		0.19		0.29
> 1800	4.5 (0.73)		3.8 (0.86)		3.8 (0.87)		3.7 (0.89)	
1201–1800	4.6 (0.61)		3.8 (0.76)		3.9 (0.72)		3.8 (0.75)	
901–1200	4.5 (0.64)		3.7 (0.78)		3.9 (0.75)		3.7 (0.79)	
≤ 900	4.6 (0.69)		3.9 (0.83)		4.0 (0.84)		3.8 (0.82)	
Previous hospitalization		0.31		0.35		0.27		0.21
Yes	4.4 (0.79)		3.9 (0.82)		4.0 (0.83)		3.8 (0.88)	
No	4.5 (0.62)		3.8 (0.75)		3.9 (0.74)		3.6 (0.79)	
QDU visits, number		0.17		0.07		0.08		**<0.001**
≤ 2	4.5 (0.54)		3.9 (0.70)		4.0 (0.68)		3.9 (0.77)	
3	4.5 (0.68)		3.7 (0.80)		3.9 (0.77)		3.7 (0.84)	
≥ 4	4.3 (0.77)		3.6 (0.88)		3.7 (0.99)		3.1 (1.05)	
Diagnosis		0.16		0.34		0.06		**<0.001**
Malignancy	4.4 (0.78)		3.7 (0.86)		3.7 (0.88)		2.9 (1.01)	
Benign disorders	4.6 (0.60)		3.8 (0.77)		4.0 (0.76)		3.8 (0.78)	

*PT* = professional training; *QDU* = quick diagnosis unit.

^a^Reverse-coded.

Bolded values are statistically significant

**Table 4:** Mean scores of clinical information, personal interaction, and recommendation subscales according to patient characteristics

Variable	Clinical information	*P* value	Personal interaction	*P* value	Recommendation	*P* value
Age, years		**<0.001**		**<0.001**		**<0.001**
18–44	3.1 (0.79)		3.0 (0.83)		8.9 (0.93)	
45–65	3.5 (0.72)		3.4 (0.73)		9.3 (0.84)	
> 65	3.9 (0.62)		3.9 (0.52)		9.8 (0.66)	
Sex		0.35		0.18		0.20
Female	3.7 (0.67)		3.8 (0.60)		9.6 (0.75)	
Male	3.8 (0.63)		3.6 (0.57)		9.4 (0.72)	
Education		**<0.001**		0.10		**<0.001**
University education	2.9 (0.86)		3.6 (0.74)		8.7 (1.22)	
Secondary/professional training	3.5 (0.77)		3.6 (0.69)		9.3 (0.86)	
Primary education	3.9 (0.64)		3.7 (0.60)		9.6 (0.72)	
No schooling	3.9 (0.66)		3.9 (0.57)		9.7 (0.74)	
Monthly income, Euros		0.22		0.15		0.17
> 1800	3.7 (0.77)		3.6 (0.75)		9.4 (0.90)	
1201–1800	3.8 (0.64)		3.7 (0.55)		9.6 (0.71)	
901–1200	3.8 (0.69)		3.8 (0.59)		9.5 (0.74)	
≤ 900	3.9 (0.73)		3.8 (0.65)		9.6 (0.80)	
Previous hospitalization		0.31		0.33		0.55
Yes	3.7 (0.76)		3.8 (0.69)		9.5 (0.82)	
No	3.8 (0.64)		3.7 (0.57)		9.5 (0.68)	
QDU visits, number		0.34		0.48		**0.002**
≤ 2	3.8 (0.59)		3.7 (0.61)		9.6 (0.67)	
3	3.8 (0.65)		3.7 (0.64)		9.5 (0.75)	
≥ 4	3.7 (0.82)		3.7 (0.73)		8.9 (1.09)	
Diagnosis		**0.007**		0.06		**0.008**
Malignancy	3.3 (0.81)		3.5 (0.72)		9.1 (0.99)	
Benign disorders	3.9 (0.63)		3.8 (0.59)		9.7 (0.69)	

*QDU* = quick diagnosis unit.

Bolded values are statistically significant

Tables 5 and 6 show the independent predictors of perceived quality of care in the physical environment, waiting times, clinical information, personal interaction, and recommendation subscales on multivariate linear regression analysis with adjusted *B* coefficients. While an age older than 65 years was a significant predictor of perceived clinical information and personal interaction and of recommendation (*B* = 0.19, 0.24, and 0.23; *P* < 0.05, <0.001, and <0.01, respectively), an age of 18–44 years was associated with lower mean scores in all three subscales (*B* = −0.18, −0.19, and −0.17, respectively; *P* < 0.05 in all). Furthermore, while no schooling was a significant predictor of clinical information and recommendation (*B* = 0.18 and 0.17; *P* < 0.05), having a university education had a strong, albeit negative, influence on mean scores of the two subscales (*B* = −0.24 and −0.22; *P* < 0.001 and <0.01, respectively). Having ≥ 4 QDU visits was associated with lower recommendation (*B* = −0.17; *P* < 0.05) and time to diagnosis scores (*B* = −0.25; *P* < 0.001). Malignancy was also significantly associated with lower time to diagnosis, clinical information, and recommendation mean scores (*B* = −0.27, −0.20, and −0.18; *P* < 0.001, <0.05, and <0.05, respectively) (Tables 5 and 6).

**Table 5:** Multivariate regression analysis of perceived quality of care in physical environment and waiting times subscales across patient characteristics

Variable	Physical environment		Time on waiting room		Visit duration		Time to diagnosis	
	*B* ^a^	95 % CI	*B* ^a^	95 % CI	*B* ^a^	95 % CI	*B* ^a^	95 % CI
Age, years								
18–44	−0.02	−0.30, 0.29	−0.03	−0.31, 0.27	−0.09	−0.39, 0.21	−0.13	−0.42, 0.16
45–65 (reference)								
> 65	0.03	−0.27, 0.32	0.01	−0.28, 0.30	0.15	−0.14, 0.45	0.16	−0.13, 0.47
Sex								
Female	−0.02	−0.11, 0.07	−0.01	−0.09, 0.11	−0.01	−0.10, 0.09	0.01	−0.08, 0.10
Male (reference)								
Education								
University education	0.01	−0.19, 0.21	−0.02	−0.21, 0.17	−0.01	−0.21, 0.18	−0.01	−0.20, 0.19
Secondary/PT (reference)								
Primary education	0.02	−0.18, 0.22	0.01	−0.17, 0.20	0.12	−0.08, 0.29	0.13	−0.08, 0.29
No schooling	0.09	−0.10, 0.28	0.03	−0.17, 0.22	0.16	−0.03, 0.33	0.12	−0.07, 0.31
Income, Euros								
> 1800	−0.09	−0.18, 0.02	0.01	−0.08, 0.10	−0.08	−0.16, 0.03	−0.07	−0.15, 0.02
1201–1800 (reference)								
901–1200	−0.09	−0.20, 0.06	−0.07	−0.19, 0.10	0.02	−0.12, 0.17	−0.08	−0.18, 0.09
≤ 900	0.02	−0.09, 0.13	0.11	−0.02, 0.24	0.12	0.02, 0.27	0.01	−0.10, 0.11
QDU visits								
≤ 2 (reference)								
3	0.01	−0.28, 0.30	−0.04	−0.38, 0.30	−0.01	−0.36, 0.34	−0.06	−0.40, 0.28
≥ 4	−0.05	−0.39, 0.30	−0.09	−0.43, 0.25	−0.10	−0.44, 0.26	−0.25^b^	−0.61, 0.12
Diagnosis								
Malignancy	−0.05	−0.44, 0.36	−0.02	−0.41, 0.37	−0.12	−0.51, 0.27	−0.27^b^	−0.68, 0.15
Benign diseases (reference)								

*CI* = confidence interval; *PT* = professional training; *QDU* = quick diagnosis unit.

^a^Adjusted regression coefficient beta. Positive values indicate a higher mean score relative to the referent category, while negative values indicate a lower mean score compared with the reference category.

^b^ *P* < 0.001

**Table 6:** Multivariate regression analysis of perceived quality of care in clinical information and personal interaction subscales and recommendation across patient characteristics

Variable	Clinical information		Personal interaction		Recommendation	
	*B* ^a^	95 % CI	*B* ^a^	95 % CI	*B* ^a^	95 % CI
Age, years						
18–44	−0.18^b^	−0.47, 0.11	−0.19^b^	−0.48, 0.10	−0.17^b^	−0.42, 0.08
45–65 (reference)						
> 65	0.19^b^	−0.09, 0.46	0.24^c^	−0.06, 0.53	0.23^d^	−0.07, 0.53
Sex						
Female	−0.02	−0.10, 0.08	0.04	−0.06, 0.15	0.12	0.02, 0.21
Male (reference)						
Education						
University education	−0.24^c^	−0.43, −0.03	0.01	−0.15, 0.20	−0.22^d^	−0.41, −0.03
Secondary/PT (reference)						
Primary education	0.16	−0.04, 0.36	0.02	−0.16, 0.21	0.10	−0.09, 0.30
No schooling	0.18^b^	−0.03, 0.39	0.11	−0.08, 0.31	0.17^b^	−0.02, 0.33
Income, Euros						
> 1800	−0.08	−0.14, 0.03	−0.06	−0.15, 0.03	−0.10	−0.2, 0.01
1201–1800 (reference)						
901–1200	0.01	−0.13, 0.14	0.10	−0.05, 0.16	−0.11	−0.25, 0.12
≤ 900	0.09	−0.04, 0.22	0.10	−0.02, 0.13	0.03	−0.09, 0.16
QDU visits						
≤ 2 (reference)						
3	0.01	−0.34, 0.35	0.02	−0.27, 0.33	−0.01	−0.35, 0.32
≥ 4	−0.02	−0.37, 0.33	0.02	−0.32, 0.40	−0.17^b^	−0.52, 0.16
Diagnosis						
Malignancy	−0.20 ^b^	−0.59, 0.21	−0.09	−0.48, 0.30	−0.18^b^	−0.57, 0.21
Benign diseases (reference)						

*CI* = confidence interval; *PT* = professional training; *QDU* = quick diagnosis unit.

^a^Adjusted regression coefficient beta. Positive values indicate a higher mean score relative to the referent category, while negative values indicate a lower mean score compared with the reference category.

^b^ *P* < 0.05.

^c^ *P* <0.001.

^d^ *P* < 0.01

## Discussion

This study presents the first detailed evidence about patient perception of quality of care of QDUs. Our results show that perceived care in all subscales and items was highly considered by most respondents. Of note, the mean score of the recommendation subscale was 9.5/10, with 81 % of respondents reporting that they would recommend the Unit ‘without a doubt’ (score of 10/10). However, regression analysis revealed that mean scores of several subscales varied significantly regarding patient age, education, number of QDU visits, and diagnosis of malignant vs. benign disorder.

Perceived quality of or satisfaction with QDUs has been poorly evaluated. In a Spanish-language descriptive study of a QDU run by internists at a Spanish second-level hospital near Barcelona, Capell *et al.* reported some data about patient opinion on the care delivered by their Unit [2]. Different samples of patients were interviewed by telephone two times, 3 months and 2 years after the introduction of QDU. The interview was performed two months after patient discharge using an in-house questionnaire with 20 questions (4 options per question), which assessed overall satisfaction, degree of difficulty of travel to hospital, and preference for type of care in subsequent episodes. The response rate was 65 and 85 % in the two study periods, respectively. The findings were similar on both occasions: while a high overall satisfaction was reported by 95 % of respondents, repeated travels to hospital were not an important difficulty, and 80 % of patients reported that they would prefer the QDU care model over conventional hospitalization should they require a new diagnostic evaluation.

These results were similar to those subsequently reported in an English-language journal [21]. In this study, whose main objective was to analyze the usefulness and costs of an internal medicine QDU compared to hospitalization in a Spanish third-level university hospital in Barcelona, a random sample of patients were interviewed by telephone 3 months after discharge. The authors used a questionnaire similar to that of Capell *et al.* [2] to evaluate similar aspects. While the response rate was 94 %, overall satisfaction with QDU was high in 93 % of cases, repeated travel to hospital was not considered a major difficulty, and 84 % of patients would choose the QDU instead of hospitalization should a further diagnostic workup be required [21]. Unlike our study, however, these former reports did not define specific subscales and constituting items and did not provide detailed results other than the overall percentages mentioned above.

The high response rate of our study (98 %) may be related to the fact that the QDU attending physician invited patients to participate in it by distributing the questionnaire in hand at the end of first visit, then reminding them to complete it at the end of last visit. While one of the most common administration methods consists of handing out questionnaires immediately after, or during, service use [28], this may result in overestimation of satisfaction (see limitations of the study below) [34].

The questionnaire selected for this study needed to be fairly brief, understandable and easy to complete. Although there are discrepancies [35], low levels of education are reportedly related to difficulties in assimilating crucial information, most notably on diagnosis and discharge instructions [36, 37], which may affect questionnaire dimensions and reported satisfaction. Seventy percent of our respondents had no schooling or primary education (55–57 % of respondents in previous Spanish hospital outpatients studies using this questionnaire [31, 32]), consistent with education data reported in a former study conducted in our hospital [20].

Perceived quality of care in all subscales was indeed high. Regarding waiting times, 69, 74 and 89 % of respondents chose the options ’short’/’very short’ or ’better’/’much better’ than expected for time to diagnosis, wait on waiting room, and visit duration, respectively. Former surveys have shown that waiting time, either perceived or real, influences satisfaction [29, 38–40]. While longer waits on waiting rooms have a strongly negative correlation with overall satisfaction [38], longer duration of the consultation time has been associated with higher levels of overall satisfaction in ambulatory practice [39, 40]. The physical environment of hospital outpatient services has also been reported to influence satisfaction [41, 42], with 94–96 % of our respondents scoring it as ’better’/’much better’ than expected.

Furthermore, while accessibility, understood as ease to find an outpatient clinic or an in-hospital ward, has been correlated with overall satisfaction [25], most respondents rated positively this subscale, understood as ease/difficulty to find the Unit and to travel frequently to hospital for outpatient visits and investigations. The fact that only 7 % of patients found repeated travels to hospital uncomfortable is reassuring since, unlike hospitalization, an objective of QDUs is that patients be able to maintain a fairly normal daily activity however serious their condition under study is (e.g. liver metastases) as long as their general health status is well enough [12, 13].

In the clinical management of patients attended in QDUs, where the likelihood of having a severe disease, particularly cancer, is often high and causes worry and anxiety to both patients and relatives (malignancy is the most common diagnosis in Spanish QDUs [14, 15, 24]), evaluating quality of care through the measurement of patient perception of physician supportiveness, empathy, and information giving is essential. Details as simple as knowing the physician name (97 % of our respondents knew it) are important because, as reported, patient receptiveness and satisfaction is higher when the physician introduces himself or herself at the time of the first encounter [31, 32, 37]. In our study, the mean scores of the subscales measuring the perception of the patient–physician encounter were high, with ≥ 90 % of respondents selecting the positive extreme ends of the items constituting the clinical information and personal interaction subscales. There is consistent evidence across healthcare settings that the most significant determinants of patient opinion of and overall satisfaction with quality of care are related to the patient-physician encounter, including interpersonal aspects of care such as an affective and trust-generating behavior (including courtesy, empathy, and supportiveness), information giving and also clinical competences/skills [28, 29, 38, 43–45]. In the case of information giving/physician feedback, overall satisfaction is negatively correlated with scant explanation of the problems and/or the examinations results and, in general, with receiving little information and answers from the medical staff [29, 38, 45–47].

An important finding of our study was that 95 % of respondents reported that they would recommend the Unit to a relative with the same disease, as determined by a score ≥7, with 81 % scoring 10/10. Although not analyzed here, the few studies that have investigated the association between perceived quality of care and willingness to recommend a given provider have revealed some interesting differences between satisfaction and recommendation [29, 48–50]. A study conducted in nearly 2000 patients in PCCs in Taiwan found that physician technical competence/skills was the most critical determinant of perceived quality for both overall satisfaction and recommendation, followed by interpersonal skills [48]. Another study conducted in nearly 5000 patients in 126 Taiwanese hospitals revealed that physician technical competence/skills was a more significant predictor of recommendation than interpersonal skills [49]. Interestingly, 21 % of the ‘not satisfied’ patients in this study still recommended the hospital, meaning that a hospital with a high number of their patients being satisfied may not receive a comparable level of recommendation. Intriguingly, in both Taiwanese studies [48, 49], the rates of ‘no answer’ responses to ‘recommendation’ questions were significantly higher that the rates of ‘no answer’ responses to ‘satisfaction’ questions, suggesting that patients may feel more responsible at the time of recommending a healthcare provider, tending to miss the relevant question when they are unsure about its quality. More recently, a multicenter study conducted in a US network of national oncology hospitals showed that perceived quality of care was also an important predictor of patient willingness to recommend the healthcare provider [50]. In this study, ‘helping a patient to understand her/his condition’, ‘caring for a patient as an individual’, ‘a whole-person approach to care’, and ‘satisfaction with the medical oncologist’ all favored patient willingness to recommend the provider. Evaluating perception of quality of care as a predictor of recommendation appears particularly important in numerous countries where data of service quality are not easily available and recommendations from relatives or friends become the essential source of information for choosing a provider [50]. Although asking patients about their intents to recommend or revisit a provider is frequently used to monitor perceived quality and satisfaction for marketing purposes, it may be argued that such methods may be less appropriate in public health services such as the Spanish one or, for instance, the UK National Health Service, where options are limited and mobility difficulties exist [29].

The most important socio-demographic predictor of satisfaction is age, with older patients being typically more satisfied with healthcare services [28, 29, 38, 45–47, 51–53]. Although studies have also found that patients with lower educational levels are in general more satisfied [28, 38, 46, 48], such association is not always clear-cut, with a number of studies reporting inverse associations (i.e. higher reported satisfaction amongst individuals with lower education attainment), something that might be related to expectations [29].

Despite the high scores observed in all subscales, there were some crucial differences associated with patient age and education on multivariate linear regression. The analysis identified age older than 65 years as an independent predictor of clinical information, personal interaction, and recommendation, while having an age of 18–44 years was significantly associated with lower mean scores in these subscales. The regression analysis also revealed that no schooling predicted higher clinical information and recommendation scores, while having a university education had marked negative influence on these subscales’ scores.

Our patients had a mean of 2.2 visits, with 60 % having ≤2 visits. The finding that having ≥4 QDU visits (vs. ≤ 2 visits) was independently associated with lower time to diagnosis and recommendation scores probably reflects the significantly longer mean time to diagnosis of respondents with ≥4 QDU visits (vs. those with ≤2 visits) [16.7 (9.94) days for ≥4 QDU visits vs. 10.8 (8.11) days for ≤2 visits; *P* < 0.001] (data not shown). Although we are not aware of studies analyzing the association between time to diagnosis and perceived quality or satisfaction in outpatients, some studies have evaluated the relationship between in-hospital length of stay (a marker of hospital efficiency and a proxy measure of cost) and satisfaction under the assumption that shorter than expected length of stay could be regarded as suggestive of good quality of healthcare, resulting in higher satisfaction levels (and vice versa) [54, 55].

Rather unexpectedly, malignancy was an independent negative predictor of time to diagnosis, clinical information, and recommendation mean scores on regression analysis. While time to diagnosis of respondents with malignancy was significantly longer than those with non-malignant diseases [15.1 (9.18) vs. 11.0 (7.67) days; *P* < 0.001] (data not shown), the significant association of malignancy with low clinical information and recommendation scores is more difficult to interpret. However, it is known that self-perceived health can be a significant predictor of satisfaction, with poorer physical health status [29, 38, 47, 51–53] and psychological distress [29, 56, 57] being associated with lower satisfaction levels. Accordingly, although not analyzed in this study, it is possible that patients with malignant conditions scored those subscales poorer as a result of a worse physical health performance associated with their disease and the psychological anguish produced by the communication of their final diagnosis, usually at the time of last visit.

Although we identified one aspect of the QDU care process that was worse than expected, patient opinion of it remained high. In particular, compared with other Spanish studies on QDUs [2, 15, 21, 22], the mean time to diagnosis was too long, reflecting delays in diagnostic tests as a consequence of variations in the organization of healthcare services in the hospital related to the Spanish financial crisis. Yet nearly 70 % of patients scored time to diagnosis as short or very short, and, on regression analysis, an age older than 65 years was close to statistical significance (*B* coefficient = 0.16; *P* = 0.07) (Table 5). This observation may illustrate the concept that it is the patient–physician encounter that mainly determines perceived quality of care and satisfaction rather that the potential patient expectation about this specific item.

It is worthy to note that the study was carried out at a time of a profound economic recession in Spain (March to December 2012), which has caused well-documented detrimental effects on the healthcare system [19, 58]. Briefly, Spain has an advanced, integrated health system that has accomplished noteworthy results, including significantly improved health outcomes, over a fairly short time. Until recently, Spain had one of the world’s highest life expectancies, an extremely low infant mortality and was a world leader in organ transplantation and donation [59]. The Spanish system was listed the 7^th^ best worldwide by the World Health Organization in 2000. While Spaniards have been traditionally proud of their system, major national and regional cuts in health spending applied as of late 2010 have not only affected the quality of care but also some health outcomes. For instance, in addition to a remarkable increase in surgical waiting lists and delays in diagnostic tests at both hospital and PCC settings, the prevalence of mental disorders, particularly major depression, and the suicide rate, has increased, mainly as a consequence of unemployment and inability to accomplish mortgage obligations [19, 58, 60]. In contrast to former national surveys revealing highly positive opinions of Spaniards on their healthcare system, a 2013 transnational survey of 12,001 adult patients from 15 countries (mainly high-income countries) showed that Spanish respondents (*n* = 1000) felt that both overall access to healthcare services (‘doctor‘,‘diagnostic tests‘,‘specialist physician‘,‘hospital‘, and ‘drugs to treat various ailments‘) and access to each specific service was more difficult in 2013 than in 2008 [61]. In fact, Spaniards rated their healthcare system as having the lowest improvement over the last five years among all countries. The questionnaire also included a question about patient experience: ‘Thinking about your patient experience recently compared to five years ago (in 2008) in going to a doctor and then being diagnosed, referred to a specialist or for surgery, or treated for an accident or serious ailment or condition, have you found it to be’. Possible answers were: ‘better information shared with me’, ‘more options given to me for treatment’, ’better quality’, ’better coordinated’, ’better level of care’, ’more sensitive to my needs’, and ’speedier’. Spaniards rating of both overall patient experience and of each category of experience compared to 5 years earlier was also the lowest among all countries. The report stated that “it is evident that respondents in Spain have seen a stark degradation in their healthcare services as they take last place in every category” of access to healthcare services and patient experience [61].

Nevertheless, cost-containment measures are leading to some sensible reforms of the system, such as the search and preferential use of alternatives to hospitalization, which is a major component of healthcare costs, including day centers, hospital at home and QDUs [12]. Although QDUs seem more proper for countries with public healthcare systems, inpatient admissions are also a major component of health costs in the USA, where PCC physicians do not facilitate non-scheduled appointments and ED physicians are more prone to hospitalize patients for a diagnostic workup [62]. As recently reported by US investigators in a systematic review of existing QDUs, “[in] our healthcare system, with the high cost of inpatient care, the QDU can yield large savings of healthcare dollars while expediting diagnostic workup, increasing patient satisfaction, and preventing lost productivity from hospital stays” [24]. One may wonder how, and to what extent, the aforementioned adverse effects of the economic crisis on the healthcare system and the reduced expectations of Spaniards about their system during the study period may relate to the observed high levels of perceived quality of care reported by our participants. Although no study has been reported on the potential influence of the financial crisis on patient satisfaction in Spain, we believe that it is precisely the essence, objectives, and reported outcomes of the QDU model that explain these results. Because of their dynamic, agile functioning and the savings generated, QDUs are currently viewed as having strong implications in Spain and possibly other countries with public health systems, where ambulatory practice is also under severe pressure. In Spain, QDUs have become a paradigm of publicly financed hospital-based outpatient units that help overcome diagnostic and referral delays and the overall difficulties faced by physicians at PCCs and EDs in ensuring quick access to investigations. Indeed, the poor coordination between primary and hospital care in Spain is reflected by the fact that, in practice, only hospitalized patients are firstly selected for prompt diagnostic examinations [12, 15, 16].

Our study has some limitations. First, the high levels of perceived quality of care including recommendation may indicate true quality of care but also reflect some methodological limitations. Thus, despite the high response rate and measures to preserve confidentiality, completing the questionnaire immediately after the visit and the fact that the QDU attending physician rather than independent researchers distributed the questionnaires may have affected the results. Studies have shown that, although asking questions in search of feedback just after the consultation has the advantage of obtaining actual time awareness, respondents tend to report more satisfaction. In contrast, although soliciting opinion excessively long after a care episode may imply that patients have forgotten relevant facts, they seem more likely to express their actual opinion when they have more time to ponder the consultation, hence tending to report less satisfaction when the questionnaire is fulfilled at home [29, 43, 63]. Anyway, a recent study concluded that there is insufficient research comparing the advantages and disadvantages of different timeframes for acquiring feedback about satisfaction or patient experience [28]. Second, the study was undertaken in a single center. Yet, according to published reports on Spanish QDUs, our patients are representative of those evaluated in other units [24]. Third, providing concurrent survey data from hospitalized patients in the same hospital for the purposes of comparison would have been desirable. Nevertheless, we intend to repeat the survey in the near future with a larger sample of QDU patients as well as to expand our investigation to include hospitalized patients. In fact, we are currently testing and validating a similar questionnaire adapted for patients who are hospitalized for diagnostic workup. Finally, our questionnaire evaluated patient perception/opinions of quality of care including recommendation rather than satisfaction. Although most questionnaires have focused on satisfaction rather than opinions about quality of care, some experts argue that the latter is a more valuable approach [26]. While perceived quality of care does not need to be expressed in terms of satisfaction [64], evaluation of satisfaction does not need to unavoidably contemplate patient opinion or views about quality of care [65]. Furthermore, besides involving a highly affective dimension [66], satisfaction seems more dependent on patient expectations than is perception of quality [67, 68].

## Conclusion

Although completion of the questionnaire after the consultation and its delivery by the QDU attending physician might have resulted in overestimation, this study shows that patients suspected of suffering a potentially severe disease attending a Spanish QDU of a third-level university hospital reported a high perception of the quality of care including waiting times, physical environment, accessibility, clinical information, personal interaction, and recommendation. Remarkably, patients’ willingness to recommend the Unit was high with a mean score of 9.5 in a visual analogue scale ranging from 0 (‘never’) to 10 (‘without a doubt’). However, multivariate linear regression analysis revealed significant differences in mean scores of several subscales with regard to patient age, education level, number of QDU visits, and a diagnosis of malignant vs. benign condition, after due adjustment. Although age older than 65 years was an independent predictor of higher clinical information, personal interaction and recommendation scores, an age of 18–44 years was associated with lower mean scores in these subscales. Furthermore, although lower education was an independent predictor of higher clinical information and recommendation scores, university education was strongly associated with lower scores in these subscales. In addition, having ≥4 QDU visits was a negative predictor of time to diagnosis and recommendation scores, likely reflecting the longer time to diagnosis of patients having more visits. Finally, malignancy was a negative predictor of time to diagnosis, clinical information, and recommendation scores. This association may be partly explained by the longer time to diagnosis of patients diagnosed with malignancy and perhaps, although not analyzed, by a worse physical health status related to their condition together with the higher anguish induced by the communication of the diagnosis at the last visit.

## Additional file

Additional file 1:
**Questionnaire Form.** Validated satisfaction questionnaire of patients evaluated in the quick diagnosis unit. (DOCX 28 kb)

## Abbreviations

ANOVA: Analysis of variance
*B*: Regression coefficient beta
CI: Confidence intervals
ED: Emergency department
PCC: Primary healthcare center
QDU: Quick diagnosis unit
SD: Standard deviation.
