# Supplementary material for: Perception of quality of care of patients with potentially severe diseases evaluated at a distinct quick diagnostic delivery model: a cross-sectional study
Source: BMC Health Serv Res. 2015 Sep 30;15:434. doi: 10.1186/s12913-015-1070-2 (PMC4589195; doi:10.1186/s12913-015-1070-2)
Supplement: Additional file 1: — Questionnaire Form. Validated satisfaction questionnaire of patients evaluated in the quick diagnosis unit. (DOCX 28 kb) [file 12913_2015_1070_MOESM1_ESM.docx]

**Questionnaire Form**

**Validated satisfaction questionnaire of patients evaluated in the quick diagnosis unit**

**Your opinion will help us improve our service. Please complete this anonymous questionnaire by choosing the answer that you think best fits your opinion**

***Please indicate the date of your first visit and your sex***:

**Date of consultation**: …/…/… **Sex**: M ^1^□ F ^2^□ ID number: …

***Please answer questions No. 1-8 when you finish the first visit and questions No. 9-20 when you finish the last visit***. ***If you only need one visit, please answer all the questions at the end of it.***

***FIRST VISIT****:*

1. **Age**: …years
2. **Education**: None ^1^□ Primary ^2^□ High school/ ^3^□ University ^4^□

Professional training

1. **Have you attended an outpatient clinic of this or another hospital?**

Yes ^1^□ No ^2^□

4**-**  **Have you ever been admitted to this hospital?** Yes ^1^□ No ^2^□

**5-** **Did you find the unit easily?**  Yes ^1^□ No ^2^□

**6-** **Do you know the name of your doctor?**  Yes ^1^□ No ^2^□

**7-** **Do you know the name of the nurse?**  Yes ^1^□ No ^2^□

**8-** **The waiting time for the visit on the waiting room was**:

Very short Short Just right Excessive Very excessive

^1^□ ^2^□ ^3^□ ^4^□ ^5^□

***LAST VISIT****:*

**9-**  **Did the healthcare staff treat you with kindness?**

Never Rarely Usually Always Not sure/don’t

remember

^1^□ ^2^□ ^3^□ ^4^□ ^5^□

**10-** **Did the healthcare staff do their best to help you when needed?**

Never Rarely Usually Always Not sure/don’t

remember

^1^□ ^2^□ ^3^□ ^4^□ ^5^□

**11-** **During the consultation, what were the following like?**

Much worse Worse As expected Better Much better

than expected than expected than expected than expected

Duration ^1^□ ^2^□ ^3^□ ^4^□ ^5^□

of the visit

Consultation ^1^□ ^2^□ ^3^□ ^4^□ ^5^□

room temperature

Noise in the ^1^□ ^2^□ ^3^□ ^4^□ ^5^□

waiting room

Consultation ^1^□ ^2^□ ^3^□ ^4^□ ^5^□

room cleanliness

**12-** **Were you given clear-cut information on the following aspects?**

Never Rarely Usually Always Not sure/don't’

remember

What your disease ^1^□ ^2^□ ^3^□ ^4^□ ^5^□

involves

The tests you ^1^□ ^2^□ ^3^□ ^4^□ ^5^□

underwent

Risks of diagnosis ^1^□ ^2^□ ^3^□ ^4^□ ^5^□

and treatment

Instructions to follow ^1^□ ^2^□ ^3^□ ^4^□ ^5^□

after discharge

**13-** **The time to diagnosis was**:

Very short Short Just right Excessive Very excessive

^1^□ ^2^□ ^3^□ ^4^□ ^5^□

**14-** **Did you have any problem with**:

Reception/personal treatment Yes ^1^□ No ^2^□ What: _______________

Diagnosis of your disease Yes ^1^□ No ^2^□ What: _______________

Medical treatment received Yes ^1^□ No ^2^□ What: _______________

Information received Yes ^1^□ No ^2^□ What: _______________

**15-** **Did you find it uncomfortable to travel repeatedly to the hospital/unit for tests and visits?**

Yes ^1^□ No ^2^□

**16-** **Would you have preferred to have been admitted to hospital to study your disease?**

Yes ^1^□ No ^2^□

**17-** **Would you recommend this Unit to a relative with the same disease, in case of need?**

0 1 2 3 4 5 6 7 8 9 10

never without a doubt

**18-** **Do you know what you have been diagnosed with?** Yes ^1^□ No ^2^□

**19-** **Please indicate your diagnosis**: ___________________

**20-** **What can we do better?** _____________________________________________________

_________________________________________________________________________
